# Quantitative estimation of chemical microheterogeneity through the determination of fuzzy entropy

**DOI:** 10.3389/fchem.2022.950769

**Published:** 2022-08-30

**Authors:** Pier Luigi Gentili, Juan Perez-Mercader

**Affiliations:** ^1^ Department of Chemistry, Biology, and Biotechnology, Università Degli Studi di Perugia, Perugia, Italy; ^2^ Department of Earth and Planetary Sciences and Origins of Life Initiative, Harvard University, Cambridge, MA, United States; ^3^ Santa Fe Institute, Santa Fe, NM, United States

**Keywords:** micro-heterogeneous chemical systems, time-resolved signals, maximum entropy method (MEM), molecular information, molecular computing, fuzzy sets, chemical artificial intelligence

## Abstract

Chemical micro-heterogeneity is an attribute of all living systems and most of the soft and crystalline materials. Its characterization requires a plethora of techniques. This work proposes a strategy for quantifying the degree of chemical micro-heterogeneity. First of all, our approach needs the collection of time-evolving signals that can be fitted through poly-exponential functions. The best fit is determined through the Maximum Entropy Method. The pre-exponential terms of the poly-exponential fitting function are used to estimate Fuzzy Entropy. Related to the possibility of implementing Fuzzy sets through the micro-heterogeneity of chemical systems. Fuzzy Entropy becomes a quantitative estimation of the Fuzzy Information that can be processed through micro-heterogeneous chemical systems. We conclude that our definition of Fuzzy Entropy can be extended to other kinds of data, such as morphological and structural distributions, spectroscopic bands and chromatographic peaks. The chemical implementation of Fuzzy sets and Fuzzy logic will promote the development of Chemical Artificial Intelligence.

## 1 Introduction

Microheterogeneity refers to systems that are heterogeneous at the microscopic level (*Kalyanasundaram, 2012*). There are numerous examples of micro-heterogeneous systems in both soft and crystalline materials (*Chen, 2022*). They might be broadly classified into five major sets (I) molecular aggregates composed of surfactants, lipids or other compounds, (II) natural and synthetic polymeric systems (III) nanomaterials and colloidal dispersions, (IV) adsorbed and intercalated guest-host solid materials (V) the multi-compartments and multiphase assemblies of living beings and their mimicries (*Cheng and Perez-Mercader, 2020*). The heterogeneity can be at the level of single particles (i.e., intra-entities) and/or inter-entities (*Chen, 2022*) (*Rabanel et al., 2019*).

No single technique can unveil all the details of these micro-heterogeneous systems. Techniques such as electron, fluorescence, Raman and atomic force microscopies, diffraction of X-rays and neutrons allow taking two- and three-dimensional snapshots of these micro-heterogeneous systems at intra- and inter-entities levels (*Rabanel et al., 2019*). Other relevant data on the collective features of micro-heterogeneous samples can be collected by techniques such as NMR and ESR. The Kirkwood-Buff theory (*Kirkwood and Buff, 1951*) describes solution mixtures containing any number of components; and it has been proven a solid framework for providing expressions of macroscopic thermodynamic features for any stable solution mixture as a function of its composition (*Pierce et al., 2008*) (*Newman, 1994*). In time-resolved spectroscopies or other techniques, transient signals monitor the dynamics and kinetics of molecular events occurring in the micro-heterogenous systems, thus providing a picture of their time evolution, including during non-equilibrium stages. The time-evolving signal I(t) of a micro-heterogeneous sample reaching an equilibrium state after a temporary perturbation or a steady-state condition when permanently maintained out-of-equilibrium can be expressed as a weighted infinite sum of exponentials, i.e.,

$$I\left(t\right)=\int_{0}^{\infty }w\left(\tau \right)e^{-t/\tau }d\tau$$ (1)

In many cases, decay kinetics from complex systems and fractal structures have been fitted by stretched exponential functions (*Berberan-Santos et al., 2005*) of the type:

$$I\left(t\right)=I_{0}e^{-{\left(\frac{t}{\tau }\right)}^{\beta }}$$ (2a)

or the less-known compressed hyperbola (or Becquerel) function (*Menezes et al., 2013*) of the type:

$$I\left(t\right)=\frac{1}{{\left(1+ct/\tau_{0}\right)}^{1/c}}$$ (2b)

However, it has been shown (*Hirayama et al., 1990*) that stretched exponential functions can be substituted by the fitting function appearing in Eq. 1. In certain conditions, a sum of compressed hyperbolas can be replaced by a sum of exponential functions (*Menezes et al., 2013*). In Eq. 1, the determination of the “image w(τ) ” (also called “eigenvalue spectrum” (*Berberan-Santos et al., 2005*)) is the inverse Laplace transform of the measured time-resolved profile (R(t)) , possibly deconvoluted from the instrument response function (IRF) , i.e., I(t) :

R(t)=(IRF)⊗(I(t)) (3)

Although deconvolution is well conditioned, inverting the Laplace transform is ill-conditioned (*McWhirter and Pike, 1978*). This implies that minor errors in the data can lead to considerable uncertainty in the reconstruction of w(τ) , a problem for which the Maximum Entropy Method (MEM) offers reliable solutions (*Jaynes, 1988*) (*Livesey and Brochon, 1987*) (*Brochon, 1994*) (*Steinbach et al., 2002*).

In the following paragraphs, the basic principles of the MEM are briefly recalled. Then, a discretization of the “image w(τ) ” is proposed along with its relationship to a discretized entropy. Such an entropy is related to the possibility of implementing Fuzzy sets through the micro-heterogeneity of chemical systems, and we therefore call it “fuzzy”. “Fuzzy Entropy” becomes a quantitative estimation of the Fuzzy Information that can be processed through micro-heterogeneous chemical systems. The chemical implementation of Fuzzy sets and Fuzzy logic promotes the development of Chemical Artificial Intelligence (*Gentili, 2021*).

## 2 The basics of maximum entropy method

The MEM’s roots reside in Bayesian probability (*Jaynes, 1957*) (*Brochon, 1994*). It provides “the least biased estimate possible on the given information; i.e., it is maximally non-committal with regard to missing information”. According to Bayes’ theorem, the probability of the hypothesis h(w) about the “image w(τ) ” of the weight coefficients appearing in Eq. 1, given the data D , is the posterior probability Pr(h|D) :

$$\mathrm{Pr}\left(h\text{|}D\right)=\frac{\mathrm{Pr}\left(D\text{|}h\right)\text{Pr}\left(h\right)}{\text{Pr}\left(D\right)}$$ (4)

The term Pr(D|h) is the “likelihood” and it represents the probability of obtaining the data D if the true “image h(w) ” is known. In the case of Gaussian noise statistics, the likelihood is:

$$\mathrm{Pr}\left(D\text{|}h\right)=\frac{e^{-\frac{1}{2}\chi^{2}}}{Z_{l}}$$ (5)

In Eq. 5,

$$\frac{1}{2}\chi^{2}=\frac{1}{2}{\left(D-Rh\right)}^{T}\left[\sigma^{-2}\right]\left(D-Rh\right)$$ (6)

with Rh being the calculated data from the “image h(w) ”, $\sigma^{-2}$ is the covariance matrix for the data, and $Z_{l}$ a normalization factor (In the case of Poisson noise, Poissonian deviance is used in the definition of the “likelihood”).

The term Pr(D) in Eq. 4 is the “plausibility” of the data based on the prior knowledge of the system. When the prior knowledge of the system remains constant, Pr(D) is like a normalization factor, $Z_{D}$ .

The term Pr(h) is the “prior probability” that represents the experimenter’s knowledge about a possible “image h(w) ” before collecting experimental data. It has an exponential form (*Gull and Daniell, 1978*):

$$\mathrm{Pr}\left(h\right)\propto e^{-\alpha S}$$ (7)

where α is a positive constant and S the Information Entropy of the true image:

S(h)=−∫h(w)log[h(w)]dw (8)

By introducing all the terms in Eq. 4, the explicit definition of the “posterior probability” Pr(h|D) is obtained as:

$$\mathrm{Pr}\left(h\text{|}D\right)=\frac{e^{\alpha S-\frac{1}{2}\chi^{2}}}{Z_{D}Z_{S}\left(\alpha \right)}=\frac{e^{Q}}{Z_{D}Z_{S}\left(\alpha \right)}$$ (9)

where $Z_{S}\left(\alpha \right)$ is a normalization factor. A solution to the inverse Laplace transform’s problem of determining w(τ) can be obtained by maximizing Pr(h|D) . The maximization of the “posterior probability” requires finding the maximum of the exponent $Q=\alpha S-\frac{1}{2}\chi^{2}$ . Q is maximized through a tug of war between the maximization of the Entropy S(h) and the minimization of the value of $\chi^{2}$ .

## 3 The discretization of the “image w(τ) ”

**FIGURE 1:**
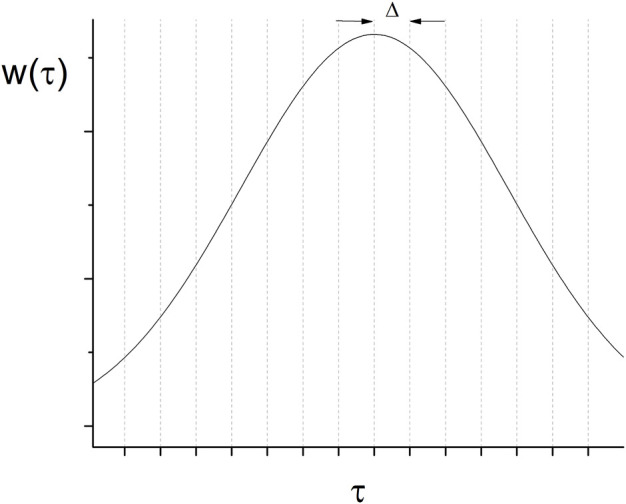
Quantization of the continuous variable τ.

A possible shape of w(τ) is shown in Figure 1. The range of τ is divided into bins of equal width Δ . It is assumed that w(τ) is continuous within the bins. Then, according to the mean value theorem (*Cover and Thomas, 2006*), there exists a value $\tau_{i}$ within each bin such that

$$w\left(\tau_{i}\right)\Delta =\int_{\left(i-1\right)\cdot \;\Delta }^{\left(i\right)\cdot \;\Delta }w\left(\tau \right)d\tau$$ (10)

The quantized variable $\tau^{\Delta }$ is introduced through the following statement:

$$\tau^{\Delta }=\tau_{i}\mathrm{if}\left(i-1\right)\cdot \Delta \leq \tau \leq \left(i\right)\cdot \Delta$$ (11)

Then, the probability that $\tau^{\Delta }=\tau_{i}$ , is (*Friar et al., 2016*)

$$p\left(\tau_{i}\right)=\int_{\left(i-1\right)\cdot \Delta }^{\left(i\right)\cdot \Delta }w\left(\tau \right)d\tau =w\left(\tau_{i}\right)\cdot \Delta$$ (12)

As Δ→0 , it is possible to approximate the integral by the sum

$$I\left(t\right)=\int_{0}^{\infty }w\left(\tau \right)\cdot e^{-t/\tau }d\tau \approx \sum_{i=1}^{N\to \infty }w\left(\tau_{i}\right)\cdot \Delta \cdot e^{-\frac{t}{\tau_{i}}}$$ (13)

If $\sum_{i}w\left(\tau_{i}\right)\;\Delta =1$ , then

$$I\left(t\right)=\sum_{i=1}^{N\to \infty }\frac{w\left(\tau_{i}\right)}{\sum_{i}w\left(\tau_{i}\right)}e^{-\frac{t}{\tau_{i}}}$$ (14)

## 4 Definition of fuzzy entropy

The information entropy of the continuous probability distribution function w(τ) is

$$H\left(w\left(\tau \right)\right)=-\int_{0}^{\infty }w\left(\tau \right)\log\left(w\left(\tau \right)\right)d\tau$$ (15)

Introducing the quantized variable $\tau^{\Delta }$ , the definition of entropy becomes:

$$H\left(\tau^{\Delta }\right)=-\sum_{i=1}^{N\to \infty }w\left(\tau_{i}\right)\cdot \Delta \cdot \log\left(w\left(\tau_{i}\right)\cdot \Delta \right)$$ (16)

$$H\left(\tau^{\Delta }\right)=-\sum_{i=1}^{N}w\left(\tau_{i}\right)\;\cdot \Delta \;\cdot \log\left(w\left(\tau_{i}\right)\right)-\sum_{i=1}^{N}w\left(\tau_{i}\right)\cdot \;\Delta \;\cdot \log\left(\Delta \right)$$ (17)

Since $\sum_{i=1}^{N}w\left(\tau_{i}\right)\cdot \Delta =\int_{0}^{\infty }w\left(\tau \right)d\tau =1$ , it follows that:

$$H\left(\tau^{\Delta }\right)=-\sum_{i=1}^{N}\frac{w\left(\tau_{i}\right)}{\sum_{i}w\left(\tau_{i}\right)}\log\left(w\left(\tau_{i}\right)\right)-\text{log}\left(\Delta \right)$$ (18)

But as w(τ)log(w(τ)) is Riemann integrable, the first term of Eq. 18 approaches the integral of −w(τ)log(w(τ)) as Δ→0 , by definition of Riemann integrability (*Cover and Thomas, 2006*). Hence, we get that $H\left(\tau^{\Delta }\right)+\log\left(\Delta \right)=H\left(\tau^{\Delta }\right)+log\left(\frac{1}{\sum_{i}w\left(\tau_{i}\right)}\right)=H\left(\tau^{\Delta }\right)-\log\left(\sum_{i}w\left(\tau_{i}\right)\right)\to H\left(w\left(\tau \right)\right)$ as Δ→0 .

The fitting procedure of the time-resolved signals through the MEM allows determining the weights $w\left(\tau_{i}\right)$ for each of the N lifetimes $\tau_{i}$ . Then, the values of the weights can be normalized:

$$\mu_{i}=\frac{w\left(\tau_{i}\right)}{\sum_{i=1}^{N}w\left(\tau_{i}\right)}$$ (19)

The variable $\mu_{i}$ can range between 0 and 1, i.e., $0\leq \mu_{i}\leq 1$ , and $\;\sum_{i=1}^{N}\mu_{i}=1$ , where N is typically greater than 100,

$$H\left(\tau^{\Delta }\right)=-\sum_{i=1}^{N}\mu_{i}\log\left(w\left(\tau_{i}\right)\right)-\log\left(\frac{1}{\sum_{i}w\left(\tau_{i}\right)}\right)=$$ (20a)

$$=-\sum_{i=1}^{N}\mu_{i}\log\left(w\left(\tau_{i}\right)\right)-\sum_{i=1}^{N}\mu_{i}\log\left(\frac{1}{\sum_{i}w\left(\tau_{i}\right)}\right)$$ (20b)

$$H=-\sum_{i=1}^{N}\mu_{i}\text{log}\left(\mu_{i}\right)$$ (20c)

The distribution of lifetimes, obtained by fitting a specific time-resolved signal, I(t) , using the MEM, allows one to implement a Fuzzy set (*Gentili, 2018*). A Fuzzy set is different from a classical Boolean set (*Zadeh, 1965*) because an element belongs to a Fuzzy set with a degree of membership $\left(\mu_{i}\right)$ that can be any real number between 0 and 1. The relative weight $\mu_{i}$ of the *i*th lifetime $\left(\tau_{i}\right)$ represents its degree of membership to the Fuzzy set of lifetimes, which granulates the time variable. The shape and position of the lifetimes’ Fuzzy set depends on the “chemical context” like any other Fuzzy set in Fuzzy logic. The output from applying the MEM gives the degree of membership $\mu_{i}$ for every lifetime $\tau_{i}$ . It is possible to determine its Fuzzy Entropy H through Eq. 20c. According to this definition, the Fuzzy Entropy H has the following two properties:

α) H=0 , if and only if we have just one lifetime, whose $\mu_{i}=1$ (i.e., the lifetime distribution looks like a crisp set).

β) H reaches its maximum value (which is log(N) ) when all the lifetimes have the same degree of membership $\mu_{i}=1/\left(N\right)$ .

Based on this second property, it is reasonable to propose the normalized version of the Fuzzy Entropy that becomes independent of the number of exponential terms used in the fitting procedure:

$$H_{nor}=-\frac{1}{\text{log}\left(N\right)}\sum_{i=1}^{N}\mu_{i}\text{log}\left(\mu_{i}\right)$$ (21)

The α  property of H also holds for $H_{nor}$ . The second property partly changes. It becomes:

β_bis_) $H_{nor}$ ranges between 0 and 1. It is 1 when all the lifetimes have the same degree of membership $\mu_{i}=1/\left(N\right)$ .

Finally, both H and $H_{nor}$ share another property:

γ) The value of Fuzzy Entropy depends on the physicochemical context of the chemical system: the more significant its micro-heterogeneity, the larger its Fuzzy Entropy.

Some experimental proofs of this third property are reported in the next paragraph. This paragraph is concluded by asserting that among the different definitions of Fuzzy Entropy that have been proposed (*Al-sharhan et al., 2001*), only that presented in Eqs. 20c and 21 is valuable for our case. The Fuzzy Entropy, appearing in Eqs. 20c and 21, is appropriate for characterizing the micro-heterogeneity of a chemical sample, based on the information retrieved by fitting any exponential time-resolved signal using the MEM.

### 5 Determination of fuzzy entropy for some chemical systems

**FIGURE 2:**
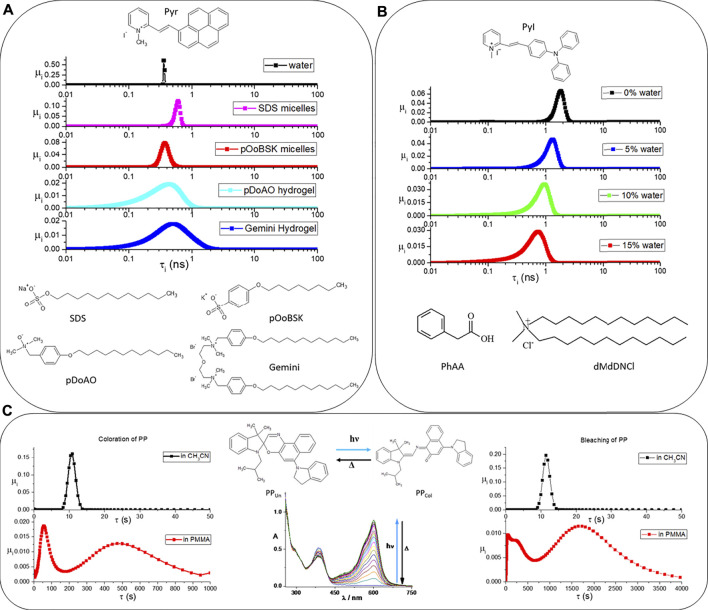
In **(A)**, the fluorescence lifetimes’ distributions of Pyr in water (black points), SDS micelles (pink points), pOoBSK micelles (red points), pDoAO hydrogel (cyan points), and Gemini hydrogel (blue points) are shown. In **(B)**, the fluorescence lifetimes’ distributions of PyI in PhAA/dMdDNCl after adding 0% (black points), 5% (blue points), 10% (green points), and 15% (red points) of water are reported. In **(C)**, the photochromism of PP (see the molecular structures of both colored and uncolored forms and the spectra recorded upon UV irradiation) has been investigated in acetonitrile and in PMMA. The plots containing the black points refer to the distributions of lifetimes for the coloration (on the left) and bleaching (on the right) kinetics in acetonitrile. The plots below, with red dots, refer to the same kinetics collected in PMMA.

The γ property of normalized Fuzzy Entropy, defined in Eq. 21, implies that $H_{nor}$ is a quantitative estimation of the micro-heterogeneity of any chemical system. Three examples supporting the validity of the statement γ are shown in Figure 2 and described hereinafter. Other proofs can be found in other works regarding biopolymers (*Comez et al., 2021*) (*Chakraborty et al., 2018*), microemulsions (*Penconi et al., 2014*), nanomaterials (*Bellacanzone et al., 2020*), dyes (*Gentili et al., 2010*) in different micro-environments, and membranes (*Krishnamoorthy and Ira, 2001*; *Haldar, 2022*)^1^.

The first example refers to the fluorescent salt 2-[(1-pyrenyl)-ethenyl]-1-methylpyridinium (Pyr). The Pyr’s fluorescent lifetimes distribution is susceptible to its micro-environment (*Cesaretti et al., 2016*). When Pyr is dissolved in pure water, the distribution is rather sharp (see Figure 2A): there are two principal components ( $\tau_{1}=0.35\;ns$ with $\mu_{1}=0.61$ , and $\tau_{2}=0.37\;ns$ with $\mu_{2}=0.37$ ) and the Fuzzy Entropy is pretty low: $H_{nor}=0.13$ . When Pyr is embedded within micelles of the anionic surfactants SDS and pOoBSK (see Figure 2A for their molecular structures), it experiences remarkably different micro-heterogeneities from that in pure water. The lifetimes’ distributions become broader, and Fuzzy Entropy becomes much larger: $H_{nor}=0.49$ and 0.55 in SDS’s and pOoBSK’s micelles, respectively. The micro-heterogeneity experienced by Pyr increases further when Pyr is dissolved in two surfactant hydrogels of intertwined wormlike micelles, made of the zwitterionic pDoAO and cationic Gemini surfactants, respectively (see Figure 2A for the molecular structures of pDoAO’s and Gemini’s surfactants). The normalized Fuzzy Entropy assumes the values of 0.80 and 0.81 in pDoAO’s and Gemini’s hydrogels, respectively.

The second example refers to another fluorescent N-methylpyridinium iodide, i.e., 2–4-(diphenylamino)phenyl-ethenyl-1-methylpyridinium iodide (PyI) used to probe the effect of water into the microheterogeneity of a Deep Eutectic Solvent (DES), which is made of phenylacetic acid (PhAA) and N,N,N,N-dimethyldidodecylammonium chloride (dMdDNCl) mixed in 2:1 M ratio (*Tiecco et al., 2021*). Figure 2B reports the fluorescent lifetimes’ distribution for PyI dissolved in PhAA/dMdDNCl and determined after addition of different amounts of water. The distribution in black (see graph on top of Figure 2B) has been determined for the DES having only its hydration water: the weighted averaged lifetime $\left({\bar{\tau }}_{av}=\sum_{i=1}^{N}\mu_{i}\tau_{i}\right)$ is 1.67 ns and $H_{nor}=0.61$ . By adding increasing amounts of water, the weighted averaged lifetime of PyI decreases progressively from 1.13 ns (after adding 5% of water) to 0.76 ns (10% of water) down to 0.59 ns (15% water). On the other hand, $H_{nor}$ grows monotonically from 0.68 (with 5% of water) to 0.73 (10% of water) up to 0.76 (15% of water). The introduction of water molecules into the hydrophobic DES determines an appreciable increase of its microheterogeneity as probed by PyI and in agreement with previous studies (*Ma et al., 2018*).

Finally, the third example is shown in Figure 2C. It regards the photochromic spiroxazine PP. When PP_Un_ is irradiated by UV, the spiro C-O bond of the oxazine is broken, and a merocyanine (PP_Col_) is produced. PP_Col_ also absorbs in the visible region. The spectral modifications that are recorded upon UV irradiation are shown in the graph below the PP’s molecular structures. Merocyanine is metastable. If UV irradiation is discontinued, spontaneous thermal bleaching of the color can be observed. The PP’s coloration and bleaching kinetics have been collected in two very different micro-environments: PP dissolved in a homogeneous solvent, such as the acetonitrile, and PP encapsulated in a micro-heterogeneous and viscous environment, such as a film of poly(methyl methacrylate) (PMMA) (*di Nunzio et al., 2010*). Both the coloration and bleaching kinetics have been fitted by poly-exponential functions through MEM. The outputs are shown in Figure 2C. They reveal that PMMA slows down the PP’s photochromism and that the polymer significantly broadens the distributions of lifetimes. The black traces, which are relatively sharp, refer to acetonitrile, whereas the red traces refer to PMMA. The values of Fuzzy Entropy quantitatively remark the differences in the lifetimes’ distributions. $H_{nor}=0.54$ and 0.46 for the colouration and bleaching of PP in acetonitrile, respectively. Such values mainly refer to the intra-entity micro-heterogeneity of PP_Col_ that exist under many conformers (*Gentili, 2014*). On the other hand, $H_{nor}=0.95$ and 0.94 for the same kinetics recorded in PMMA. The kinetic properties of PP are strongly affected by the degree of the micro-heterogeneity encompassing the PP’s molecules. Such high values of $H_{nor}$ includes both the intra- and inter-entities microheterogeneity for PP in PMMA.

## 6 Discussion

This work proposes an approach for quantitatively determining the degree of micro-heterogeneity of any chemical sample. Our approach requires, at first, the acquisition of a time-resolved signal that can be fitted by a poly-exponential function. Then, the least number of exponential terms and their relative weights are determined through the MEM. The relative weights are then used to calculate the normalized Fuzzy Entropy $H_{nor}$ according to Eq. 21. The $H_{nor}$ value becomes a quantitative estimation of micro-heterogeneity. It might refer to micro-heterogeneity at the intra- and inter-entities level: it depends on how the original time-resolved signal was originated. When comparing $H_{nor}$ determined for distinct samples and from data collected in different laboratories, the signal-to-noise ratio must be pondered since noise can affect the width of the lifetimes’ distributions (*Steinbach, 2002*).

It is interesting to note that the definition of $H_{nor}$ can also be applied to other kinds of data including morphological and structural distributions, spectroscopic bands or chromatographic peaks.

Any compound will exhibit different $H_{nor}$ values depending on its physicochemical context. Any context-dependent distribution of a particular variable becomes a way for implementing a Fuzzy set. The complete granulation of a variable will require a system of adequately chosen chemical compounds. Such a system might be the fundamental ingredient for implementing a Fuzzy Logic System. It will allow processing Fuzzy logic as it was accomplished by *Gentili et al. (2016)* after granulating the UV and visible regions through the absorption bands of properly chosen photochromic compounds. This approach allows encoding a chemical language in ways alternative to those already proposed by *Dueñas-Díez and Perez-Mercader (2019)*.

## 7 Methods

In this paper, we point out that there exists a way for quantifying micro-heterogeneity. The approach we propose is synthetically the following one.

First, we collect a transient signal generated by our sample in its environment (for instance, a luminescence decay after photo-excitation).

Second, we fit the transient signal using the Maximum Entropy Method (MEM). Then, MEM gives us the least number of exponential terms needed to describe the experimental signal (please, see paragraph 2). A weight $w\left(\tau_{i}\right)$ is associated with each lifetime $\tau_{i}$ .

Finally, we propose the Normalized Fuzzy Entropy, expressed through Eq. 21, to quantitatively determine the micro-heterogeneity which is then based on the weight $w\left(\tau_{i}\right)$ values.

## Data Availability

The original contributions presented in the study are included in the article, further inquiries can be directed to the corresponding author.

## Footnotes

1 We remind the reader that De Luca and Termini (*1972*) were the first to propose a definition of the entropy $H^{\prime }$ of a Fuzzy set. Then, many other definitions have been proposed (*Singh and Sharma, 2019*; *Prakash et al., 2008*; *Bathia et al., 2013*). These definitions have been utilized in many applications (*Bathia et al., 2015*): for instance, in the field of machine learning for features selections in pattern recognition problems. They assure that the Fuzzy Entropies of a Fuzzy set and its complement are equivalent. This condition does not have any physicochemical meaning because the chemical implementation of a Fuzzy set based on the states of a single compound cannot simultaneously include the above and the potential states associated with the complement. Therefore, the contribution of the complement is excluded from the definition proposed in Eq. 21 for quantitatively characterizing chemical micro-heterogeneity. This is akin to when in chemical kinetics concentrations are always greater than zero.
